# The Scaling Relationships between Leaf Mass and Leaf Area of Vascular Plant Species Change with Altitude

**DOI:** 10.1371/journal.pone.0076872

**Published:** 2013-10-11

**Authors:** Sha Pan, Chao Liu, Weiping Zhang, Shanshan Xu, Nan Wang, Yan Li, Jing Gao, Yang Wang, Genxuan Wang

**Affiliations:** 1 The state key laboratory of plant physiology and biochemistry, Institute of Ecology, College of Life Sciences, Zhejiang University, Hangzhou, China; 2 Key Laboratory of Plant and Soil Interactions, Ministry of Education, College of Resources and Environmental Sciences, China Agricultural University, Beijing, China; University of Nottingham, United Kingdom

## Abstract

The scaling relationship between leaf dry mass and leaf surface area has important implications for understanding the ability of plants to harvest sunlight and grow. Whether and how the scaling relationships vary across environmental gradients are poorly understood. We analyzed the scaling relationships between leaf mass and leaf area of 121 vascular plant species along an altitudinal gradient in a subtropical monsoon forest. The slopes increased significantly with altitude, it varied from less than 1 at low altitude to more than 1 at high altitude. This means that plants growing at high altitude allocate proportionately more biomass to support tissues in larger leaves and less in smaller leaves, whereas the reverse is true at low altitude. This pattern can be explained by different leaf strategies in response to environmental pressure and constrains.

## Introduction

Leaf dry mass (M) and leaf surface area (A) are two important leaf traits of the vast majority of vascular plants [1]. The relative changes in these two parameters can be described as a ‘power law’, mathematically taking the form: M = βA^α^, where β is the normalization constant and α is the scaling exponent [2], [3]. This formula reveals that SLA, the light-capturing surface built by the plant per unit investment of dry mass, is size-dependent. Since SLA = A/M and M = βA^α^, it follows that SLA = (1/β)A^1-α^. The value of α>1 indicates that larger leaves have lower SLA than smaller ones, whereas α<1 means the opposite. If M scales isometrically to A (i.e. α = 1), then changes in leaf size have no impact on SLA. Quantifying the scaling relationships between leaf size and SLA will improve our understanding of how leaves maintain a positive carbon balance and influence whole plant fitness.

Ecologists have reported empirical evidences with respect to SLA and leaf size. Several studies have shown that as leaves increase in mass, increases in surface area often fail to keep pace with the increases in mass (i.e. α>1) [2], [4], which has been called “diminishing returns”. Alternatively, it has been noted that SLA increased with leaf size, which yields “increasing returns” (i.e. α<1) [4]–[6]. Both phenomena probably occur due to different biomass distribution between productive and support tissues in large compared to small leaves [7]–[9]. Additionally, it was also found that leaf mass scales isometrically to leaf area (i.e. α = 1) [4], [10], this is size-independent and results in a “break even” relationship.

Within the leaves, there are at least two components: an expanded lamina (i.e. productive tissues) and a beam-like petiole (i.e. support tissues) [11]. Leaf biomass partitioning is an important driver of whole-plant net carbon gain. Plant growth rate scales positively with the mass fraction in leaf lamina and is negatively associated with the fraction of support tissues [7]. Some researches indicate that leaf size modifies the distribution of leaf biomass between productive and support tissues [2], [4], which further leads to the underlying allometric scaling relationships between M and A (i.e. α>1 or α<1).

Environmental factors may influence the relationship between M and A. Plant modularity has allowed plant to optimize resource distribution among different structures [12]. Optimal allocation theory predicts that plants should invest more biomass to the compartment that acquires the most limiting resource to adapt to environmental changes [13]. For example, in some extreme environments such as strong wind, compared to smaller leaves, larger leaves may increase the proportion of biomass allocation to lamina support tissues to provide sufficient mechanical stability [14]. Plants adapted to such unfavorable environments will exhibit M–A slopes>1 (i.e. diminishing returns). Furthermore, M–A slopes would vary with the environmental gradients as leaves adopt different biomass allocation strategies in response to environment changes. Prior studies have examined the M-A scaling relationships among and within species [2], [4], [10], and whether and how the scaling relationships vary across environmental gradients are still poorly understood.

Along altitudinal gradients, the environment changes rapidly over short distances. Plants subject to lower temperature, higher irradiance and strong wind at higher altitude, while light limits at lower altitude. Herein we investigated the effects of altitude on the scaling relationship between M and A in a subtropical monsoon forest. The specific questions we asked were (i) What are the relationships between M and A at different altitudes? (ii) Whether the slope of the log M–log A relationship change with increasing elevation, and if so, how?

## Materials and Methods

### Ethics Statement

This research was approved by the Administration Bureau of Mt. Tianmu Nature Reserve in China.

### Study Site

This study was conducted in Mt. Tianmu Nature Reserve, the north subtropical area of eastern China (30° 19.61′–30° 28.90′ N, 119° 25.67′–119° 26.41′ E). The altitude varies from 350 m to 1506 m. Mean annual precipitation ranges from 1390 mm at lower elevations to 1870 mm at higher elevations, and mean annual air temperature decreases from 14.8°C at the foot of the mountain to 8.8°C at the top. The reserve has obvious vertical vegetation zones, including evergreen broad-leaved forest (350–850 m), evergreen and deciduous broad-leaved mixed forest (850–1100 m), deciduous broad-leaved forest (1100–1380 m), and dwarf forest (1380–1506 m).

### Sampling

Six collection sites were chosen at ca. 200 m elevation intervals along the elevation in May 2012 (Table 1). A total of 121 vascular plant species were sampled. At each site, the most abundant plant species were selected. For each species, leaves were gathered from at least three different adult individuals which are similar-sized and not shaded by neighboring plants, and then bulked into a single sample. Petioles were included in leaf area and mass measurements. The petioles in simple-leaved species were assumed to be analogous to the sum of petioles, rachises, and petiolules in compound-leaved species [7]. Leaf area was measured with a leaf area meter (CI-203, Laser leaf area meter CID, Inc. USA). Dry mass was determined after oven-drying at 70°C for at least 72 h.

**Table 1:** Scaling exponents (α) and intercepts (β) of M-A relationship at six altitudes as estimated by RMA regression.

Altitude (m)	Latitude (N°)	Longitude (E°)	α	95% CI of α	β	95% CI of β	R^2^	Sample size
414	119°26.41′	30°19.61′	**0.859** ^c^	0.780, 0.946	−2.103	−2.249, −1.957	0.823	78
620	119°26.24′	30°19.90′	0.963^bc^	0.853, 1.088	−2.248	−2.463, −2.033	0.642	97
850	119°26.00′	30°20.24′	1.000^b^	0.912, 1.097	−2.374	−2.541, −2.206	0.704	138
1086	119°26.03′	30°24.49′	1.113^ab^	0.974, 1.273	−2.473	−2.541, −2.206	0.640	81
1286	119°25.67′	30°20.64′	**1.299** ^a^	1.160, 1.455	−2.868	−3.114, −2.623	0.773	72
1462	119°25.51′	30°28.90′	**1.258** ^a^	1.161, 1.364	−2.821	−2.993, −2.649	0.839	99

All regressions were significant (*P*<0.05). Boldfaced slopes are statistically significant different from 1 at *P*<0.05 level. The Post-hoc multiple comparison of slopes were shown among altitudes, where the slopes sharing the same superscript letters are not significantly different from each other at *P*<0.05 level.

### Statistical Analysis

The data for A and M were log-transformed. Since functional rather than predictive relationships were sought [15], reduced major axis (RMA, also called standardized major axis) regression was used to determine the scaling exponent and constant of log–log linear functions. Differences in the regression slopes among altitudes were tested by multiple post hoc comparisons. The significance level for testing slope heterogeneity and difference from slope = 1 was P<0.05. All of the analyses were conducted using SMATR Version 2.0 [16].

## Results

**Figure 1:**
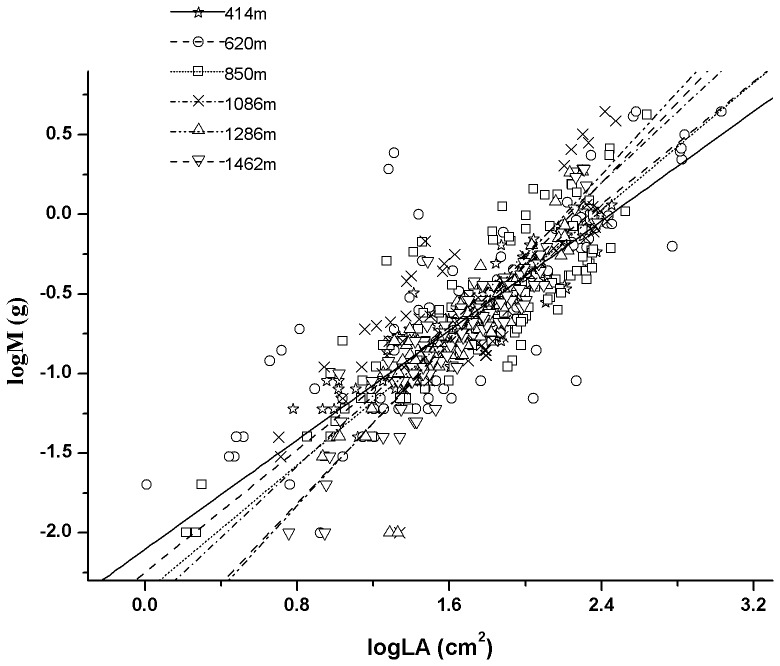
Leaf mass-area relationship at six sites as estimated by RMA regression.

**Figure 2:**
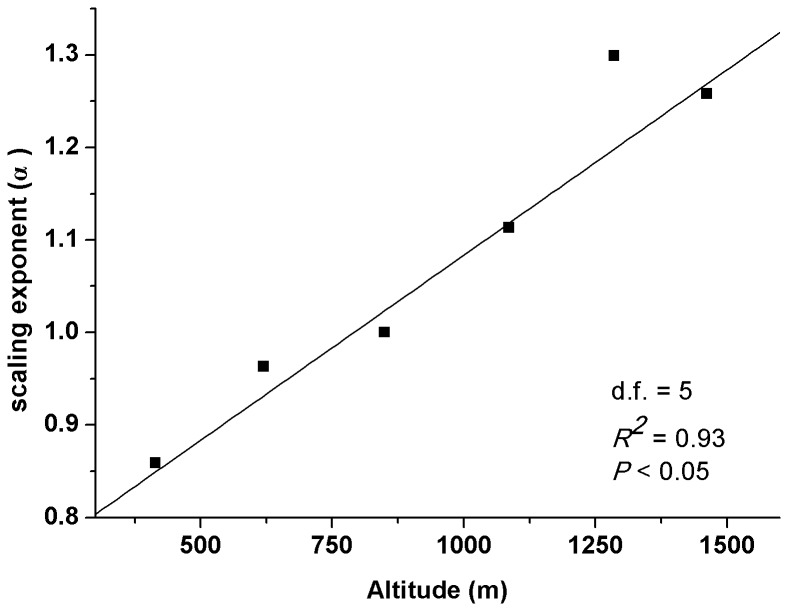
Scaling exponent (α) of the leaf mass-area relationship increases with altitude.

There was significant positive relationship between leaf mass and leaf area at each altitude (Table 1, Fig. 1). The slopes showed a great degree of variability among altitudes, ranging from 0.859 to 1.299. The slopes were significantly<1 at low altitude (414 m), whereas significantly>1 at high altitudes (1286 m and 1462 m). In middle altitudes, the slopes were not significantly different from 1 (620 m, 850 m and 1086 m). The *post hoc* multiple comparisons of slopes among altitudes showed that slopes at high altitudes were significantly higher than those at low altitude (Table 1). Furthermore, there was a highly significant positive relationship between the estimated slopes and altitude (Fig. 2).

## Discussion

The M-A scaling relationship has important implications for understanding the ability of plants to acclimate to environmental conditions [17]. In the present study, we found a wide range of variation in M to A scaling exponents among altitudes (from 0.859 to 1.299) (Fig. 1, 2). This is consistent with results from prior theoretical and empirical studies in which no constant value was used to describe the M-A relationships across all leaves [2], [12], [17]. Environmental controls on specific leaf area induces the variant leaf allometry [9]. Leaves are subject to strong selective gradients in aridity, solar radiation, and nutrient availability that affect their size and shape [12], [18]. Accordingly, the scaling values are expected to vary across environments as they balance the need for efficient conductance, net carbon acquisition, and protection against desiccation.

The scaling of M vs. A affects leaf economy in size-dependent ways, which has an important implication for leaf size optimization [11]. In this study, M scales ‘faster’ than A, and larger leaves show lower SLA than smaller ones at high altitudes (i.e. α>1) (Fig. 1, 2). Plant performances are limited by low temperature, high irradiance and strong wind at high altitudes. Leaf support structure provides laminas with both mechanical support and a pathway for water and nutrient transport. Low temperature would limit the transportation efficiency and thus, leaves may require a high investment in the transporting structure [11]. High irradiance and strong wind would increase the proportion of biomass allocation to leaf support structure [19]–[21]. In particular, large leaves require rigid support and mechanic resistance because they suffer large drag forces and static loads [20], [22]. Therefore, large leaves tend to have a larger fractional biomass investment in support structure relative to small ones. Small leaves, on the other hand, produce smaller wind-induced drag forces and have lower support needs, and thus a higher fraction of productive tissue. This diminishing return in the scaling of leaf size with leaf support investment implies that small leaves with greater SLA are more likely to be favoured at higher altitudes.

In our study, SLA increases disproportionately with increasing A at low altitude (i.e. α<1) (Fig. 1, 2). Light is a key limiting resource for plant growth and survival at low altitude [23]. Plants grown under low light intensities tend to have low photosynthetic capacities per unit leaf area [24], [25], thus, plants would evolve to maximize the biomass allocation to laminas and minimize the lamina support investment to capture more light. Leaf size has important consequences for the scale and precision with which plants forage for light. Large leaves at low light intensities may intercept a large amount of light due to their more extensive foliar display, whereas small leaves are better able to exploit fine-grained environmental heterogeneity by positioning their leaves in light-rich micro-patches [26]. It has also been reported that leaf shape, leaf angle and petiole length alter leaf light-interception efficiency [27]. Small leaves regular their leaf arrangement to fills the gaps via modifications in petiole length and thus to take advantage of the penumbra effect. Hence, more investment in petioles may radically change light-interception capacity of small leaves. However, efficient light harvesting via supporting structural modifications may become increasingly expensive with increasing leaf size [7]. Large leaves may manifest an adaptive modification towards avoiding enhanced costs for leaf support, and may consequently construct cheaper and more extensive light-intercepting foliar display than small leaves. Thus, plants may optimise a pay-off of having large leaves and efficient light intercepting surface and high SLA with low investment of photosynthesising tissues per unit area at low altitude. Environmental conditions at middle altitudes are in between low and high altitude, which thus lead to the isometric relationship between M and A (i.e. α = 1) (Fig. 1, 2). Our results imply that the differences in size-dependent SLA may be an adaptive response to limits imposed on plant growth and survival by environmental conditions.

The scaling relationship reflects the results of an evolutionary trade-off among many ancestral metabolic, morphological, and anatomical traits shared by all vascular plants [2]. Our research here exclusively focused on the altitude gradient, and therefore in-depth understanding of the developmental mechanisms underlying allometric strategies across other environmental gradient (e.g., aridity) requires further exploration.
